# Treatment and counselling preferences of postpartum women with and without symptoms of (childbirth-related) PTSD: findings of the cross-sectional study INVITE

**DOI:** 10.1186/s12884-024-07061-2

**Published:** 2024-12-31

**Authors:** Rosa Hannele Horstmann, Lara Seefeld, Julia Schellong, Susan Garthus-Niegel

**Affiliations:** 1 Institute and Policlinic of Occupational and Social Medicine, Faculty of Medicine, TUD Dresden University of Technology, Dresden, Germany; 2 Department of Psychotherapy and Psychosomatic Medicine, Faculty of Medicine and University Hospital Carl Gustav Carus, TUD Dresden University of Technology, Dresden, Germany; 3 Institute for Systems Medicine (ISM), Faculty of Medicine, Medical School Hamburg, MSH, Hamburg, Germany; 4 Department of Childhood and Families, Norwegian Institute of Public Health, Oslo, Norway

**Keywords:** Postpartum, PTSD, Childbirth-related PTSD, Help-Seeking, Preferences, Treatment and counselling services, Service provision mode, INVITE study

## Abstract

**Background:**

Post-traumatic stress disorder (PTSD) in the postpartum period is a prevalent yet under-researched mental health condition. To date, many women who suffer from postpartum PTSD remain unrecognized and untreated. To enhance the accessibility of help for these women, it is crucial to offer tailored treatment and counselling services that align with their needs. This study aimed to understand how support preferences differ between women with and without postpartum PTSD, considering the two subgroups of postpartum PTSD: childbirth-related PTSD (CB-PTSD) and general PTSD (gPTSD).

**Methods:**

This study used data from the cross-sectional INVITE study, comprising telephone interviews with *N* = 3,874 women conducted six weeks to six months after childbirth. The City Birth Trauma Scale (City BiTS) was used to assess CB-PTSD, while the Primary Care Posttraumatic Stress Disorder Screen for DSM-5 (PC-PTSD-5) was used to assess gPTSD. Service preferences and preferred modes of service provision were measured with self-developed questionnaires. Analyses of variance were used to identify differences between groups.

**Results:**

The support services *(family-)midwives* and *family, friends, or colleagues* and the service provision mode as *in person communication* were preferred most by women across groups. The analyses revealed that women with CB-PTSD had lower overall preferences for services compared to women without postpartum PTSD. Women with CB-PTSD also showed less preference for psychotherapeutic services (e.g. outpatient treatment, inpatient clinics) compared to women without postpartum PTSD. Regarding modes of service provision, women with gPTSD had a higher preference for all service modes compared to women with CB-PTSD and those without postpartum PTSD, with a stronger preference for both direct (e.g. in person, video conference) and delayed communication (e.g. chat, e-mail).

**Conclusion:**

This study was the first to explore the support preferences of women experiencing symptoms of postpartum PTSD. Findings suggest that women differ in their preferences, contingent upon the subgroup of postpartum PTSD. According to women's overall preferences, the expansion and further training of (family-)midwife services is recommended. By tailoring support services accordingly to women’s preferences, it may be possible to bridge the treatment gap for postpartum PTSD and to improve the well-being of affected women and their families.

## Background

Childbirth is a transformative and profound experience, celebrated by society as a joyous event. However, for a significant proportion of women, it can also be a source of overwhelming distress. This distress can manifest in various forms, including postpartum depression (PPD) and postpartum anxiety disorder (PAD) being the most prevalent and best studied [1–4].

Childbirth-related posttraumatic stress disorder (CB-PTSD) may be another form of postpartum distress that remains less well studied [5], despite its significant prevalence. Recent meta-analyses estimate that it affects 4.7% of women in community samples [6] and up to 18.5% in high-risk groups [7], highlighting the need for further research. The Diagnostic and Statistical Manual of Mental Disorders—5th edition (DSM-5) outlines PTSD as a severely disabling condition characterised by intrusive thoughts, avoidance of trauma reminders, negative cognitions and mood, and hypervigilance following a traumatic event [8].

Up to one in three women experience childbirth as traumatic [9–11]. These traumatic birth experiences can for instance include unexpected birth events such as emergency caesarean section or preterm birth, which can result in the development of CB-PTSD. Potential risk factors for the development of CB-PTSD include antenatal vulnerabilities, such as mental health problems and fear of childbirth, as well as risk factors during childbirth, such as a negative subjective birth experience, lack of support during delivery, and obstetric interventions [12–14].

While the awareness of CB-PTSD in research and clinical practice seems to have increased in recent years, there is still a lack of recognition that postpartum trauma-related symptoms may be caused by events other than childbirth. General PTSD (gPTSD) can be present both before and after childbirth as a result of traumatic events such as physical violence, sexual abuse, or accidents during a woman's lifetime. However, distinguishing between CB-PTSD and gPTSD may prove difficult, because childbirth can also act as a potential trigger or exacerbating factor for PTSD symptoms resulting from prior traumatic experiences, such as sexual abuse. As a result, research has rarely considered these different subgroups of postpartum PTSD as distinct subgroups, although there is evidence for differences in clinical features and phenomenology, such as more re-experiencing in CB-PTSD compared to more avoidance in gPTSD [15]. Prevalence rates also appear to differ between the two subgroups in the postpartum period, with post-traumatic stress resulting from an event other than childbirth being more common, with a prevalence rate of 6.8% for gPTSD compared to 2.5% for CB-PTSD found in a British sample [15, 16].

**Fig. 1:**
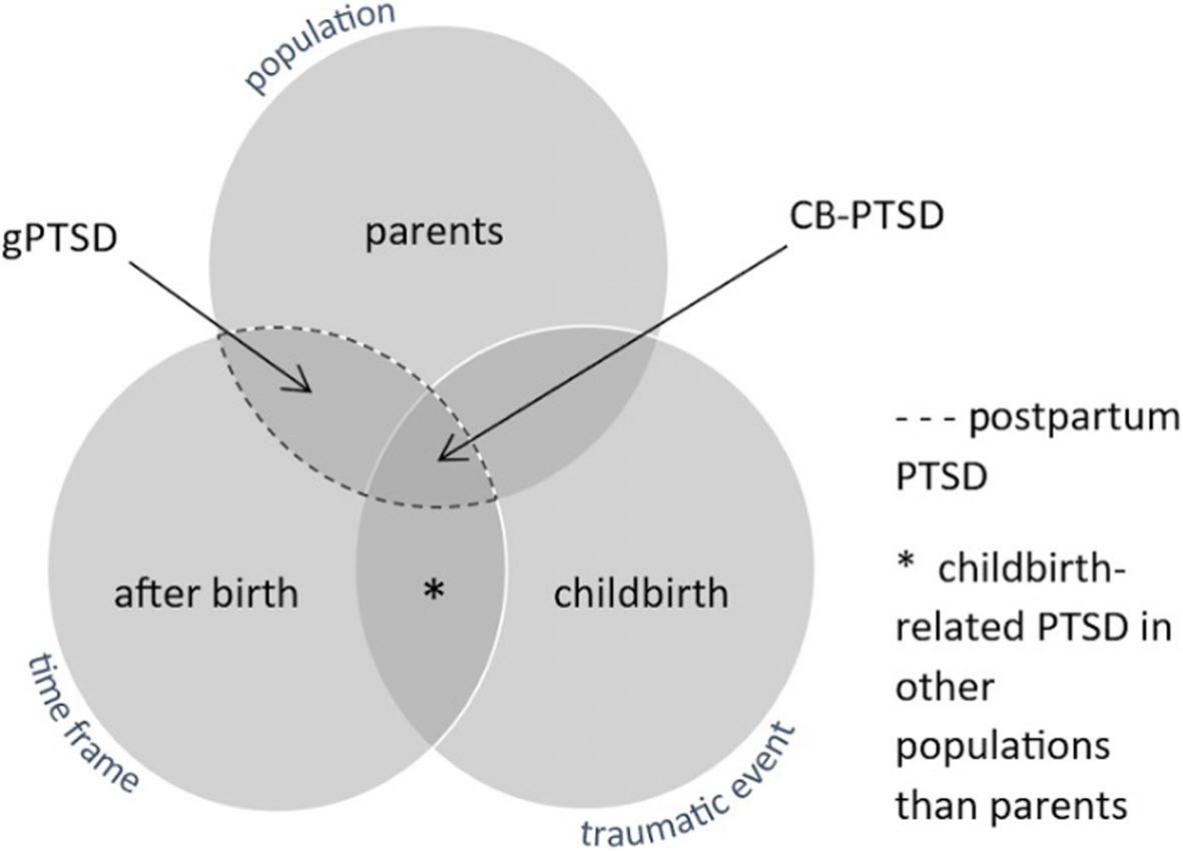
Representation of CB-PTSD and the differentiation of the constructs gPTSD and postpartum PTSD (Unpublished observation. Marlena Harder, Lara Seefeld, Julia Schellong, Susan Garthus-Niegel, 2024). Note. Definitions and abbreviations used in this work for the symptom groups of childbirth-related PTSD (CB-PTSD) and general PTSD (gPTSD), collectively referred to as postpartum PTSD. Adopted from Harder et al. [18] based on the model proposed by Heyne et al. [6]

Besides such differences, CB-PTSD has the unique status of being a mental health condition that results from an event that is typically perceived as positive. This supposedly positive image of the event childbirth becomes crucial when questioning the awareness of post-traumatic distress in our society, particularly within the healthcare system, and the willingness of women to report post-traumatic distress and seek the necessary support [17]. Therefore, it is important to distinguish between the two subgroups of CB-PTSD and gPTSD, collectively referred to as postpartum PTSD (see Fig. 1), when studying women who experience post-traumatic distress after childbirth.

**Fig. 2:**
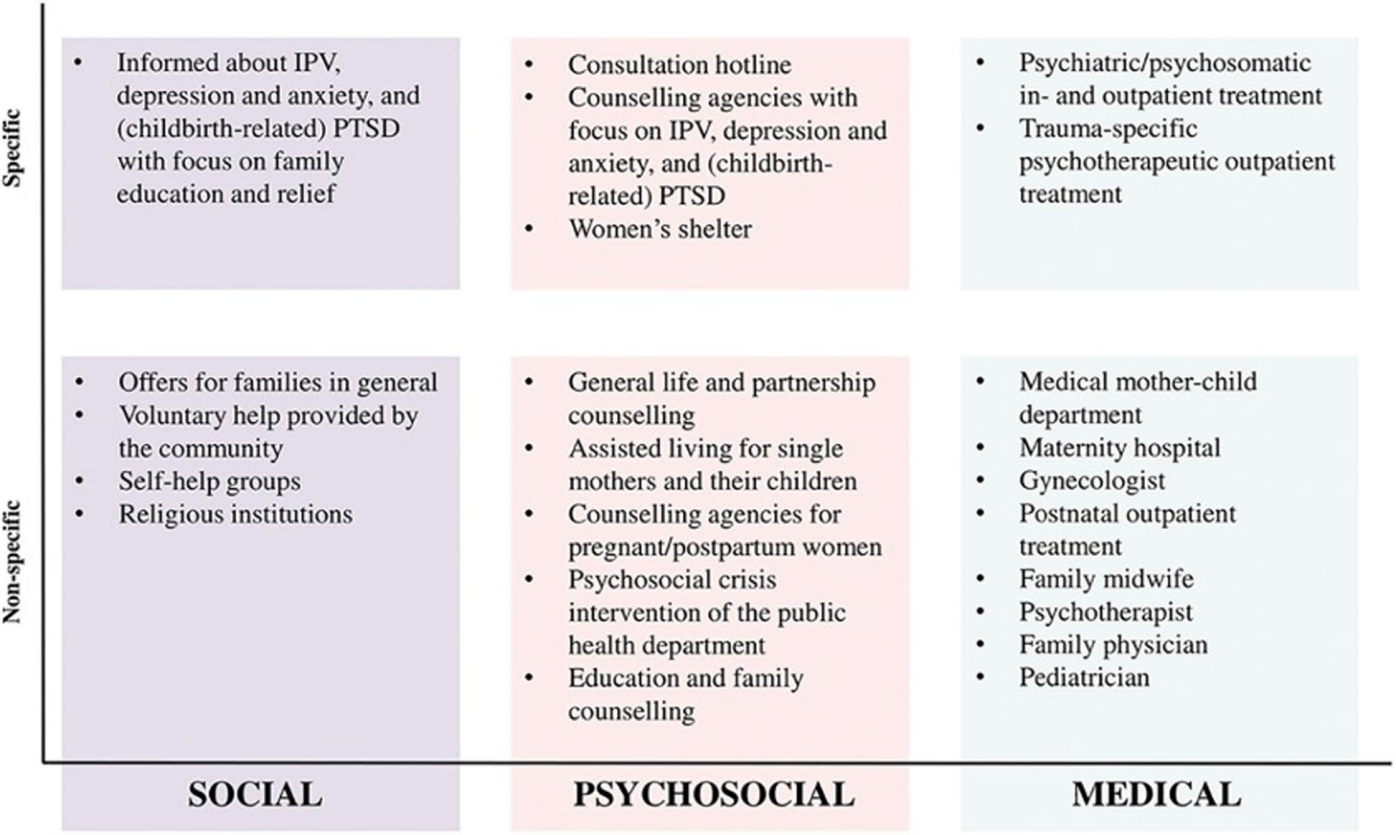
Treatment and counselling structures of the German support system (Seefeld et al., 2022) [30]. Note. Non-specific and specific treatment and counselling services available in the social, psychosocial, and medical domains of the German support system. IPV = intimate partner violence. From “Preferences and Barriers to Counseling for and Treatment of Intimate Partner Violence, Depression, Anxiety, and Posttraumatic Stress Disorder Among Postpartum Women: Study Protocol of the Cross-Sectional Study INVITE “ by L Seefeld, A Mojahed, F Thiel, J Schellong and S Garthus-Niegel, 2022, *Frontiers in Psychiatry* 13:83635. 10.3389/fpsyt.2022.836350. Copyright 2022 by Lara Seefeld. Reprinted with permission

The symptoms and adverse effects associated with postpartum PTSD can be effectively addressed and enhanced through existing interventions as evidenced by the findings of research studies [19–21]. However, women who suffer from postpartum PTSD may not receive adequate support due to a lack of awareness among health professionals and frequent misdiagnosis of postpartum PTSD as PPD [22–24]. Moreover, barriers such as fear of stigma or logistical reasons (e.g. not having childcare) can prevent women from seeking help [25, 26]. Health experts have emphasised the need for women-centred care services for many years [27–29] and the German support system provides a variety of social, psychosocial, and medical services for postpartum women, which include both mental health-specific and non-specific options, see Fig. 2. At present, however, there is a paucity of research examining which of these service options is most preferred by women with postpartum PTSD.

Qualitative research indicates that women with CB-PTSD primarily seek emotional support from informal sources such as family and friends [31, 32]. Besides, peer support also appears to be crucial for mothers with mental health problems in the postpartum period, counteracting the isolation and social pressure often associated with these conditions [33]. One notable example of a professional service in the context of CB-PTSD is birth debriefing, an intervention women seek to feel valued and to understand their birth [34]. Birth debriefing is popular intervention in maternity services, particularly within the UK. However, there is a paucity of empirical evidence substantiating its efficacy in improving PTSD symptoms [20, 35]. This gap highlights a need for further research to inform current practice. Women who seek professional help for mental health problems after childbirth highlight the need for trauma-informed care approaches [32] and emphasise the importance of trust and consistency in their interactions with healthcare providers, as well as an open and non-judgmental attitude [31, 36]. Midwives are one important professional group for women when experiencing mental health problems after childbirth and regular, prolonged contact can be an effective approach for the establishment of the desired trust and consistency. (Family-)Midwives^1^and gynaecologists were found to be the most favourable professional service providers for women with PPD and PAD [37]. Considering that PPD and PAD often co-occur with CB-PTSD [38], it is plausible that women experiencing these mental health disorders have similar preferences. This overlap in preferences may stem from shared symptomatology, such as negative perceptions of the self and others in both PPD and PTSD [8]. This shared symptomatology can lead to more negative attitudes towards seeking help [39], which may act as a barrier to accessing support services. A more profound comprehension of the discrepancies in attitudes among women with postpartum mental health conditions could help to enhance the accessibility and efficacy of interventions for this group.

However, differences in these attitudes are also possible. For CB-PTSD specifically, it is plausible that affected women may exhibit a markedly negative attitude towards medical treatment options due to commonly reported negative experiences with medical professionals during childbirth [40]. Although there are indications of possible service preferences among women with postpartum PTSD, there is currently a lack of quantitative research on women's preferences across social, psychosocial, and medical domains, especially considering possible differences between women with CB-PTSD, gPTSD, and women without clinically relevant symptoms of postpartum PTSD. It is important to note that women who do not meet the full criteria for postpartum PTSD may still experience significant distress, affecting their well-being and ability to care for their newborn. Early intervention can prevent exacerbation of symptoms, so it is important to address the lack of care for these women, too.

In addition to various treatment and counselling options, there are also different modes by which these services can be provided. These modes of service provision involve contact in person, by telephone, and video calls, as well as chat and app-based options. To improve access to care for women with postpartum PTSD, it is important to provide treatment and counselling in modes that meet women's needs.

Telehealth in the treatment and counselling of mental health issues has been on a rise in recent years and can be provided in real-time communication (e.g. via telephone, video conference) as well as asynchronously in delayed communication (e.g. via e-mail, chat, app-based) [41]. As an example in the context of psychotherapy, delayed communication may include delivering therapy modules online, with therapist support provided through follow-up messages [42] or online-structured writing therapy [43]. Studies confirm the effectiveness of telehealth intervention in the treatment of PTSD, similar to that of in person interventions [44]. They offer high-quality care with expertise for particular conditions regardless of geographical distance and can help overcome barriers that often prevent individuals from seeking help [41]. A barrier for postpartum women may be finding childcare to attend an appointment, which telehealth options could help overcome [45]. Yet the acceptance for telehealth services was found to be low for women with PPD when compared to in person services [46, 47]. It is questionable whether women with postpartum mental health problems will find the desired trusting relationship with health professionals, as mentioned above, in modes of service provision other than face-to-face contact.

Women’s preferences in mode of service provision and whether women with CB-PTSD or gPTSD and women without clinically relevant symptoms of postpartum PTSD differ in their preferences remains to be studied.

To summarize, most of the existing research on women's preferences for postpartum mental health support has been qualitative in nature [25, 31, 32]. Comprehensive quantitative analyses with large sample sizes are needed to identify effective ways of supporting women with postpartum PTSD to access appropriate treatment and counselling services. This will help to bridge the gap between women's health care needs and services available in the support system. An understanding of the support preferences of women with postpartum PTSD and those without clinically relevant symptoms is particularly needed to develop tailored and effective approaches to postpartum care, as differences in preferences may provide targets for intervention. At the same time, it is important to recognise that women without clinically relevant symptoms of postpartum PTSD, i.e. women who do not meet the full criteria for postpartum PTSD, may still experience some distressing symptoms and may benefit from treatment and counselling as well. Their preferences should therefore also be considered.

### Research questions

This study aims to provide insights into the support preferences of women with and without postpartum PTSD, understanding that women without postpartum PTSD may not meet the clinical thresholds but may still experience subthreshold symptoms. The following research questions will be investigated: Do women with symptoms of CB-PTSD, gPTSD, and women without clinically relevant postpartum PTSD symptoms differ in (1) their overall rating of available treatment and counselling services, (2) their preferences for specific services, (3) their overall ratings of service provision mode, or (4) their preferences for specific modes of service provision? Given the current lack of studies providing evidence on group differences in support preferences between women with CB-PTSD, women with gPTSD, and women without clinically relevant postpartum PTSD symptoms, these research questions will be tested exploratively.

## Methods

The data collection for this paper was done as a part of the cross-sectional study **INVITE **(**IN**timate partner **VI**olence care and **T**reatment pr**E**ferences in postpartum women). This paper used data from Version 4 of the quality-assured datafiles from the INVITE study, collected between the study initiation in November 2020 and July 2023.

### Design

The objective of the INVITE study was to gain insights into the experiences of postpartum women affected and unaffected by PPD, PAD, (CB-)PTSD, and intimate partner violence (IPV), focusing on their preferences and barriers to treatment and counselling. In the present study, we conducted a comprehensive analysis of the INVITE data set of women interviewed thus far, evaluating the construct of interest in relation to our research question on the preferences of women with and without postpartum PTSD. The data were collected through structured telephone interviews conducted by trained student assistants. These interviews comprised various quantitative questionnaires to assess the constructs of interest. All questionnaires were answered during the telephone interview, with the interviewers asking the women the questions verbally so that they could answer directly. Following the one-hour telephone interview, all women were provided with a list of treatment and counselling options available in Dresden. As an incentive for participation, women received 20 euros in total. A comprehensive description of the INVITE study can be found in the published study protocol by Seefeld et al. [30].

Data collection and management was facilitated using Research Electronic Data Capture (REDCap), a secure, web-based application for data capture as part of research studies, hosted at “Koordinierungszentrum für Klinische Studien” at the Faculty of Medicine of the Technische Universität Dresden [48, 49]. The INVITE study was reviewed and approved by the Ethics Committee of the Technische Universität Dresden (No: EK 139042016).

### Sample

The study focused on a community sample of postpartum women in the Dresden area in Germany. Inclusion criteria were childbirth within the last 6 – 26 weeks and sufficient language skills in German or English.

**Fig. 3:**
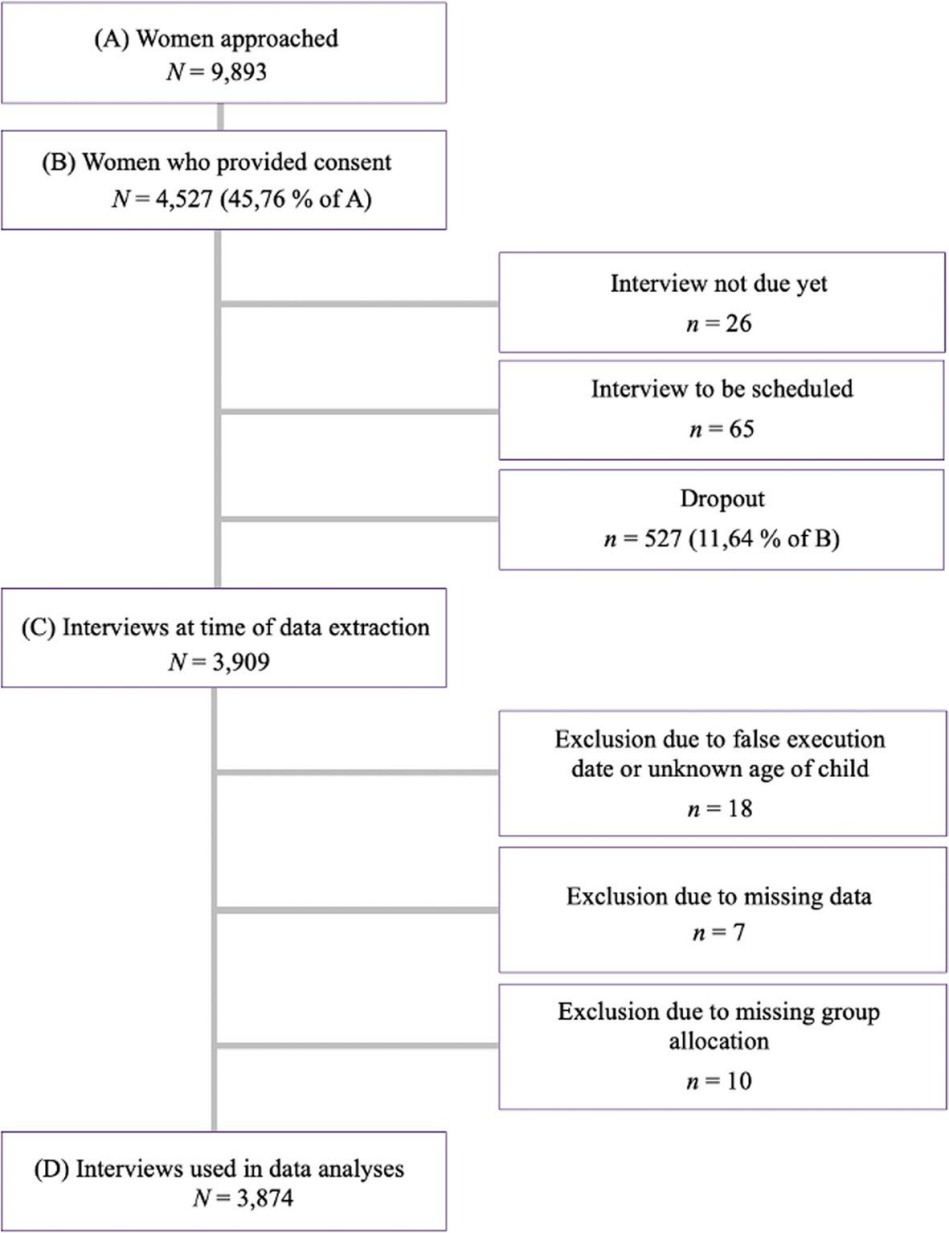
Flowchart of study population and retention rate. Note. Illustration of the response rate, dropouts, exclusions, and final sample size of this work based on the recruitment process of the INVITE study between November 2020 and July 7th, 2023

Recruitment was conducted at all maternity units in Dresden, as well as most birthing centres. Midwife appointments and birth information events at the hospitals served as additional recruitment channels. Women were mostly approached by student assistants. A total of 9,893 women were approached and informed about the study of whom 4,527 (45,76%) provided written consent. At the time of data extraction, 527 participants (11,64%) dropped out of the study. Additionally, for 26 women, the interview was pending and for 65 women an interview appointment still needed to be arranged, as they had not yet fallen within the designated period for the interview, which was 6 – 26 weeks after birth. This resulted in a total of 3,909 completed interviews at the time of data extraction. For this work, 18 women were excluded due to interviews taking place outside of the designated period (6 – 26 weeks after birth), 7 women were excluded due to missing data in all relevant constructs and another 10 women were excluded due to missing data in group specific constructs leading to missing group allocation. Consequently, the final sample included in this study comprised *N* = 3,874 women. Figure 3 illustrates the recruitment process and the retention of the study population.

## Materials

The study used established and validated instruments to answer the research questions whenever possible, in addition to self-developed questionnaires.

### City Birth Trauma Scale (City BiTS)

Symptoms of CB-PTSD were assessed employing the German version of the City BiTS, a 29-item questionnaire designed to measure CB-PTSD based on DSM-5 criteria [9, 50]. The stressor criterion (i.e. threatened death or serious injury of the mother or baby during birth or immediately afterwards) was assessed by two dichotomous items (*yes/no*). The key symptoms of intrusion, avoidance, negative cognitions/mood, and hyperarousal were covered by 22 items. These symptoms' frequency within the week prior to assessment was rated on a four-point Likert scale from 0 (“*never*”) to 3 (“*5 or more times*”). The severity of symptoms was illustrated by the total score of 0 – 60, with higher scores indicating a higher level of CB-PTSD symptoms. Five additional items evaluated the onset and duration of symptoms, impairment and distress, as well as potential exclusion criteria [50].

The internal consistency of the City BiTS in our sample was high, as indicated by Cronbach’s alpha (⍺ = 0.84). Previous research has confirmed convergent validity of the City BiTS with other PTSD instruments (Impact of Event Scale – Revised (IES-R) and Posttraumatic Stress Disorder Checklist (PCL-5)), and divergent validity with the depression (Edinburgh Postnatal Depression Scale (EPDS)) and anxiety instruments (Depression Anxiety Stress Scales (DASS-Anxiety)) [50].

### Primary Care Posttraumatic Stress Disorder Screen for DSM-5 (PC-PTSD-5)

To assess PTSD symptoms related to an event other than childbirth, a short version of the PC-PTSD-5, a screening instrument for PTSD according to DSM-5 criteria, was used [51, 52]. Previous research has confirmed the validity of the PC-PTSD-5 through high correlation with the Clinician-Administered PTSD Scale for DSM-5 (CAPS-5) [52]. For our study, the PC-PTSD-5 was adjusted to ask participants to first think of an event that was *“so frightening, horrible, or upsetting”* that they experienced any of the five key PTSD symptoms within the past month. This adjustment according to Harrison et al. [15] allowed the presence of potential PTSD symptoms to be captured for all women, not just those who reported experiencing an index event according to the DSM-5 criterion A. The presence of each of the five key symptoms was assessed using yes/no questions, resulting in a PC-PTSD-5 score ranging from 0 – 5. The reliability of the score was considered acceptable according to Cronbach's alpha (⍺ = 0.73). If a symptom was present, participants were asked how distressing they experienced it on a five-point Likert scale ranging from 0 (*“not at all”*) to 4 (*“extremely distressing”*). Perceived distress due to PTSD symptoms was quantified on a scale from 0 – 20, with higher scores indicating increased distress and thus greater symptom severity. Participants were then asked to report, in a multiple response format, any traumatic events they had experienced other than childbirth-related events (e.g. serious life-threatening illness, sexual assault) [53].

### Assessment of treatment and counselling service preferences

Preferences for treatment and counselling services were evaluated using a self-generated questionnaire based on Simhi et al. [47]. The questionnaire covered 20 treatment and counselling options divided into four subcategories: personal and professional confidants (e.g. family, friends, midwives), communal and psychosocial services (e.g. counselling centres), medical services (e.g. gynaecologist, general practitioner), and psychotherapeutic services (e.g. day clinic, psychotherapeutic practice) [37]. Services were specifically chosen to cover all available options of the German support system, as illustrated in Fig. 2, provided for women with postpartum mental health problems in the area of Dresden [30]. Participants were asked whether they would use a service if they are, or imagine they were, suffering from symptoms of postpartum mental health problems on a four-point Likert scale from 1 (“*not at all*”) to 4 (“*definitely*”). This provided a total score (20—80) for the overall preference for all service options as well as scores for the four subscales. The internal consistency of the total score of treatment and counselling service preferences was good, as indicated by Cronbach's alpha (⍺ = 0.80). The internal consistency of the subscales was evaluated using McDonald's ω, which revealed an unacceptable level of consistency for professional and personal confidents (ω = 0.44), a questionable level for medical services (ω = 0.66), an acceptable level for communal and psychosocial services (ω = 0.77), and a good level for psychotherapeutic services (ω = 0.82). Further background information on the questionnaire and the calculation of the subscales based on principal factor analysis with varimax rotation can be found in Zieß et al., 2024.

Participants were additionally asked whether they would prefer a mother–child therapy or a therapy of the mother alone for psychosomatic or psychiatric treatment options.

### Assessment of preferred mode of service provision

A self-generated questionnaire was used to assess the preferred mode of service provision with 7 items [30]. The questionnaire comprised two subscales, one for direct modes of service provision (real-time communication; i.e. telephone call or video conference) and one for delayed modes (asynchronous communication; i.e. via e-mail or chat) [37]. The service provision mode ‘in person’ was excluded from the subcategory ‘direct modes of service provision’ due to insufficient discriminant power, as determined in a previous study that initially analysed the questionnaire of preferred mode of service provision by principal factor analysis with Varimax rotation [37]. For a detailed description of how the subscales of the questionnaire were derived, please refer to the work by Zieß et al. 2024. Women were asked to what extent each mode of service provision meet their needs. Answers were rated on a four-point Likert scale from 1 (“*not at all*”) to 4 (“*definitely*”) resulting in a total score (7—28). Internal consistency was acceptable for the total score of service provision mode preference (⍺ = 0.71). McDonald's ω indicated acceptable internal consistency (ω = 0.78) for the delayed communication subscale. As the subscale for direct communication consisted of two items, reliability was assessed using inter-item correlation (*r* = 0.415, *p* < 0.001), in line with the recommendation of Clark et al. [54].

## Data analysis

IBM SPSS Statistics (version 29.0.0.0) was used for data analysis. Descriptive analyses in terms of mean and standard deviation were conducted on sample characteristics and outcome variables.

As shown in previous research, specific sociodemographic variables such as maternal age, duration of residence in Germany, income, and parity can have an impact on preferences for postpartum support [47, 55–57]. Pearson correlation analysis between those potential covariates and the outcome variables was performed. Only variables that showed a significant correlation (*p* < 0.05) with the outcome variables (sum score of service preferences and sum score of mode of service provision preferences) were included in the respective analyses as covariates.

To address the research questions, analyses of group differences were conducted between three groups: women with CB-PTSD, women with gPTSD, and women without clinically relevant symptoms of postpartum PTSD or PTSD at all. The CB-PTSD group included women who met the diagnostic criteria in the City BiTS [9], without necessarily meeting the full criterion A, but having experienced PTSD like symptoms. The theoretical rationale for not applying the criterion A to the CB-PTSD group is for the fact that even objectively non-threatening birth experiences can be subjectively experienced as traumatic by women [58]. The gPTSD group included women who experienced a stressor event other than childbirth and had a PC-PTSD-5-score of ≥ 4 [59]. The group of women without clinically relevant postpartum PTSD symptoms did not meet the threshold for CB-PTSD or gPTSD on either scale. A fourth group of comorbid women who met criteria for both CB-PTSD and gPTSD were evaluated descriptively, as inferential analyses could not be performed due to the small size of this group.

Two one-way analyses of covariance (ANCOVA) were used to examine group differences in preferences for treatment and counselling services as well as preferences for mode of service provision (total scores). Two one-way-multivariate analyses of covariance (MANCOVA) were used to compare group differences in the sub scores of the preferences of treatment and counselling services and the preferences of service provision modes. In case of significant group differences (*p* < 0.05), post-hoc-tests using Bonferroni-corrected mean comparisons were conducted for the ANCOVAs and MANCOVAs.

All assumptions for the analyses have been checked. Assumptions were either given or balanced by robust methods. There were small variations in group sizes of women included in the analyses because of missing values for the variables of interest. Missing values of less than 20% in one scale were replaced by the women’s mean value.

### Power analysis

To determine the required sample size, an a priori power analysis was performed using G*Power 3.1.9.6 [60]. For F-tests (ANCOVA), the power analysis calculated a total sample size of *n* = 246 participants required to detect small effects (*d* = 0.2) with 80% (1—β) power and an alpha value of 0.05. G*Power does not provide a power analysis for MANCOVA but using the calculated sample size for MANOVA to detect small effects (*n* = 600, *f* ^2^ = 0.01, 80% power, α = 0.05) and considering a much larger expected sample size in the current data, we inferred high statistical power for the intended MANCOVA analyses.

## Results

### Descriptive analyses

The final sample comprised *N* = 3,874 women. Their descriptive characteristics are presented in Table 1. Most of the mothers, with a mean age of 32.95 (*SD* = 4.64) years, were born in Germany (91.7%), currently in a partnership (97.7%), and reported a high level of education, defined as more than 10 years of education (74%). Their newborns were on average 13.18 (*SD* = 2.71) weeks old. In this sample, 93.4% of women were without clinically significant symptoms of postpartum PTSD, while 3.7% of women met criteria for CB-PTSD and 2.5% of women met criteria for gPTSD. A group of *n* = 15 women reported symptoms of comorbid CB-PTSD and gPTSD but was excluded from further group comparison analyses as the statistical power would have been too low.

**Table 1:** Sample characteristics

Characteristics	*M (SD)*	Range
**Maternal age**^**a**^	32.95 ± 4.64	16.78 – 53.96
**Age of newborn**^**b**^	13.18 ± 2.71	6.00 – 26.00
	***n***	**%**
**Partnership status**		
Partner	3785	97.7
No Partner	89	2.3
**Duration of residence in Germany**^**c**^		
Born in Germany	3532	91.2
< 5 years	103	2.7
5 – 10 years	115	3.0
> 10 years	124	3.2
**Education**		
≤ 10 years	1008	26.0
> 10 years	2864	74.0
**Income**^**d**^		
< 1,250€	93	2.4
1,250€ – 2,249€	397	10.3
2,250€ – 2,999€	493	12.7
3,000€ – 3,999€	1001	25.9
4,000€ – 4,999€	960	24.9
> 5,000€	914	23.7
**Parity**		
Primiparous	2029	52.4
Multiparous^e^:	1845	47.6
1	1415	76.7
2	332	18.0
3	74	4.0
≥ 4	23	1.2
**Symptom groups**		
without ^f^	3618	93.4
CB-PTSD	143	3.7
gPTSD	98	2.5
Comorbid CB-PTSD and gPTSD	15	0.4

*City BiTS* City Birth Trauma Scale, *PC-PTSD-5* Primary Care Posttraumatic Stress Disorder Screen for DSM-5

^a^n years

^b^in months

^c^time since potential migration to Germany

^d^net income per household and month

^e^additional children to latest newborn

^f^without clinically significant symptoms of postpartum PTSD

The order of preference for treatment and counselling services as well as the preference for mode of service provision among all women is shown in Table 2. The most popular services were *midwives, family midwives,* and *family members/friends/colleagues*, while *religious institutions* were the least preferred. In terms of mode of service provision, most women showed a preference for *in person communication*, followed by *video conference* and *telephone calls*. The least popular option was service provision via an *app or online platform without expert guidance*.

**Table 2:** Preferences of treatment and counselling services and service provision mode across all groups

Treatment and counselling service preferences	*M*	*SD*
1. Midwife	3.65	0.60
2. Family midwife	3.46	0.71
3. Family member, friend, or colleague	3.45	0.76
4. Woman in the same situation	3.30	0.75
5. Gynaecologist	3.27	0.79
6. Outpatient clinic/treatment for psychiatry, psychosomatic medicine, or psychotherapy	3.16	0.77
7. Life and family counselling centre	3.10	0.76
8. General practitioner	3.07	0.87
9. Specialized trauma outpatient clinic	3.03	0.78
10. Paediatrician	2.88	0.95
11. Household help	2.86	0.92
12. Supervised parent group	2.75	0.80
13. Psychosocial crisis service	2.74	0.81
14. Self-help group	2.70	0.86
15. Day clinic for psychiatry or psychosomatic medicine	2.63	0.86
16. Social pedagogical family assistance	2.55	0.86
17. Inpatient clinic for psychiatry or psychosomatic medicine	2.37	0.89
18. Telephone counselling	2.30	0.85
19. Parent–child living or family accommodation	2.24	0.97
20. Religious institutions	1.75	0.85
**Mode of service provision preferences**		
1. In person	3.70	0.53
2. Video conference	2.60	0.84
3. Telephone call	2.50	0.75
4. App or online platform with guidance from expert	1.96	0.77
5. E-mail	1.81	0.75
6. Chat	1.75	0.73
7. App or online platform without guidance from expert	1.50	0.62

Women were asked which type of therapy they would prefer for psychotherapeutic treatment options. The results are presented in Table 3. Women without clinically relevant postpartum PTSD symptoms, women with CB-PTSD, as well as women with gPTSD all indicated a preference for mother–child therapy over therapy of the mother alone. This trend was most pronounced in the group of women without clinically relevant postpartum PTSD symptoms and less so in the CB-PTSD and the gPTSD groups. Only women in the comorbid group preferred the therapy of the mother over mother–child therapy.

**Table 3:** Preference for type of psychotherapeutic treatment

Treatment type	without^a^	CB-PTSD	gPTSD	Comorbid CB-PTSD and gPTSD
	*n* (%)			
Therapy of the mother	1325 (36.6)	72 (50.3)	44 (44.9)	11 (73.3)
Mother–child therapy	2297 (63.5)	81 (56.6)	55 (56.1)	5 (33.3)
Don't know	364 (10.1)	4 (2.8)	9 (9.2)	0 (0)

Both types of treatment could be chosen

^a^without clinically significant symptoms of postpartum PTSD

Descriptive results for the predictor variables (City BiTS score and PC-PTSD-5 score) and for the outcome variables (preferences for treatment and counselling and preferences for service provision mode, including the total sum scores and the subscale scores) of women by symptom group are presented in Table 4.

**Table 4:** Descriptive statistics of predictor and outcome variables

Predictor / outcome variable		without^a^	CB-PTSD	gPTSD	Comorbid CB-PTSD and gPTSD
	*M (SD)*				
City BiTS^b^		5.05 ± 0.84	19.92 ± 0.61	10.68 ± 0.91	25.13 ± 2.45
PC-PTSD-5^c^		0.37 ± 0.01	0.92 ± 0.09	4.34 ± 0.05	4.33 ± 0.13
**Treatment and counselling service preferences**					
** Total sum score**		55.60 ± 0.123	53.89 ± 0.614	54.23 ± 0.79	53.07 ± 2.41
Professional and personal confidants^d^		13.88 ± 0.03	13.58 ± 0.16	13.65 ± 0.17	13.67 ± 0.46
Communal and psychosocial services^e^		21.26 ± 0.07	20.78 ± 0.36	20.98 ± 0.42	18.87 ± 1.31
Medical services^f^		9.25 ± 0.03	8.97 ± 0.17	8.80 ± 0.23	9.27 ± 0.68
Psychotherapeutic services ^g^		11.22 ± 0.04	10.57 ± 0.22	10.78 ± 0.31	11.27 ± 0.71
**Mode of service provision preferences**					
** Total sum score**		12.05 ± 0.05	11.68 ± 0.25	12.96 ± 0.37	11.07 ± 0.82
Direct communication^h^		5.08 ± 0.02	4.83 ± 0.10	5.42 ± 0.15	4.53 ± 0.38
Delayed communication^i^		6.97 ± 0.04	6.84 ± 0.19	7.55 ± 0.28	6.53 ± 0.58

*City BiTS* City Birth Trauma Scale, *PC-PTSD-5* Primary Care Posttraumatic Stress Disorder Screen for DSM-5

^a^without clinically significant symptoms of postpartum PTSD

^b^City BiTS score (0 – 60)

^c^PC-PTSD score (0 – 5)

^d^including family member, friend, colleague, women in the same situation, midwife, family midwife

^e^including social pedagogical family assistance, parent–child living or family accommodation, psychosocial crisis service, life and family counseling center, supervised parent group, telephone counseling, self-help group, household help

^f^including pediatrician, gynecologist, general practitioner

^g^including day clinic for psychiatry or psychosomatic medicine, outpatient clinic/treatment for psychiatry, psychosomatic medicine, or psychotherapy, inpatient clinic for psychiatry or psychosomatic medicine, specialized trauma outpatient clinic

^h^including telephone call, video conference

^i^including e-mail, chat, app, or online platform with guidance from expert, app or online platform without guidance from expert

### Correlation analysis

To define the covariates to be included in each analysis, Pearson correlation analysis was performed with all potential covariates and outcome variables. Statistically significant correlations were found for maternal age, income, and duration of residence in Germany with the outcome service preference sum score as well as mode of service provision preference sum score (*p* < 0.05). Therefore, maternal age, income, and duration of residence in Germany were included in both ANCOVAs and both MANCOVAs. Parity was only found to be significantly correlated with one subscale of service preference (personal confidents) and was therefore not included in the respective analyses. For the detailed correlation matrix, see Table 5.

**Table 5:** Pearson correlation matrix including all potential covariates and outcome variables

	1	2	3	4	5	6	7	8	9	10	11	12
1. Maternal age	-											
2. Income	.208**	-										
3. Residence in Germany	.033**	.051**	-									
4. Parity	.297**	.056**	.044**	-								
5. Service preference sum	.054**	.061**	.037*	.016	-							
6. Services Subscale 1^a^	-.004	.134**	.167**	-.062**	.535**	-						
7. Services Subscale 2^b^	.044**	.005	-.016	.031	.846**	.283**	-					
8. Services Subscale 3^c^	.057**	.007	-.022	.014	.477**	.195**	.156**	-				
9. Services Subscale 4^d^	.045**	.067**	.030	.026	.735**	232**	.475**	.197**	-			
10. Mode preference sum	.042**	.033*	-.057**	-.026	.180**	.088**	.180**	.066**	.105**	-		
11. Modes Subscale 1^e^	.073*	.116**	.008	-.029	.213**	.139**	.187**	.054**	.163**	.705**	-	
12. Modes Subscale 2^f^	.011	-.026	-.082**	-.016	.110**	.033*	.127**	.055**	.042**	.906**	.338**	-

Significant correlations between covariates and outcome variables (treatment and counselling service preferences sum score and mode of service provision sum score) are printed in bold. *p < .05. **p < .01

Potential covariates = Maternal age, income, duration of residence in Germany, parity

^a^Personal confidants

^b^Communal and psychosocial services

^c^Medical services

^d^Psychotherapeutic services

^e^Direct communication

^f^Delayed communication

### Group comparison for treatment and counselling service preferences

#### Total sum score

As indicated by a one-way ANCOVA, the symptom groups differed significantly in the total sum score of preferences for counselling and treatment services (F(2, 3811) = 3.808, *p* = 0.02, partial η^2^ = 0.002). The partial η^2^ indicated a very small effect size according to Cohen. Bonferroni-corrected post-hoc tests revealed significant differences between women with CB-PTSD and women without clinically relevant symptoms of postpartum PTSD (*p* = 0.034, *M*_*Diff*_ = 1.591, 95% CI [−3.096, −0.085]), but not for the other symptom groups (see Table 6). Women with CB-PTSD preferred all treatment and counselling service options less (*M* = 53.89, *SD* = 0.61) than did women without clinically relevant postpartum PTSD symptoms (*M* = 55.60, *SD* = 0.12), see Table 4.

**Table 6:** Group differences in the total sum score of treatment and counselling service preferences

(I) Comparing symptom groups	(J) Comparing symptom groups	Mean difference (I-J)	*p*	95% CI	
				LL	UL
without^a^	CB-PTSD	**1.591**	.034*	.085	3.096
	gPTSD	.921	.699	-.938	2.770
CB-PTSD	without^a^	**−1.591**	.034*	−3.096	-.085
	gPTSD	-.670	1.000	−3.011	1.672
gPTSD	without^a^	-.921	.699	−2.770	0.928
	CB-PTSD	0.670	1.000	−1.672	3.011

*CI* Confidence interval, *LL* Lower limit, *UL* Upper limit

Significant group mean differences are printed in bold

^a^without clinically significant symptoms of postpartum PTSD

*p< .05

#### Subscales

To examine group differences in the preferences for specific subcategories of treatment and counselling services, a one-way MANCOVA was computed, which revealed significant group differences (F(8, 7506) = 2.006, *p* = 0.042 partial η^2^ = 0.002, Wilk’s Λ = 0.996) in the subscale of psychotherapeutic services (F(2, 3756) = 5.685, *p* < . 003, partial η^2^ = 0.003), indicating very small effect sizes according to Cohen [61]. There were no other significant differences between groups on the other subscales of treatment and counselling service preferences. Post-hoc tests using Bonferroni-corrected means showed that women with CB-PTSD rated psychotherapeutic services less favourably than women without clinically relevant postpartum PTSD symptoms (*p* = 0.007, *M*_*Diff*_ = −0.691, 95% CI [−1.236, −0.146]), see Table 7. As the assumption of homogeneity of regression slopes between the symptom groups for the covariates maternal age and German residence was not fulfilled for one subcategory each, their interaction terms were included in the MANCOVA. Maternal age showed significance in the subscale of psychotherapeutic services. A descriptive analysis revealed that in the gPTSD group, older women had a greater preference for psychotherapeutic services, whereas this effect was less pronounced or absent in the group of CB-PTSD and women without clinically relevant postpartum PTSD symptoms (results not pictured). The covariate duration of residence in Germany showed significance in the subcategory of medical services. A descriptive analysis suggested that longer duration of residence in Germany is associated with a greater preference for medical services in the gPTSD group. In contrast, the CB-PTSD group showed a decrease in preference for medical services with a longer duration of residence in Germany (results not pictured). These associations between covariates and women's preferences in the subcategories imply trends rather than verified statistical effects and should be interpreted with caution.

**Table 7:** Group differences in the sub scores of preferences for psychotherapeutic services

(I) Comparing symptom groups	(J) Comparing symptom groups	Mean difference (I-J)	*p*	95% CI	
				LL	UL
Psychotherapeutic services					
without^a^	CB-PTSD	**.691**	.007**	.146	1.236
	gPTSD	.018	1.000	-.676	.713
CB-PTSD	without^a^	**-.691**	.007**	−1.236	-.146
	gPTSD	-.673	.192	−1.542	.197
gPTSD	without^a^	-.018	1.000	-.713	.676
	CB-PTSD	.673	.192	-.197	1.542

*CI* Confidence interval, *LL* Lower limit, *UL* upper limit

Significant group mean differences are printed in bold

^a^without clinically significant symptoms of postpartum PTSD

^*^p < .05, **p < .01

### Group comparison for preferences for mode of service provision

#### Total sum score

The mean preferences score of mode of service provision differed significantly among the groups (F(2, 3810) = 3.690, *p* = 0.025, partial η^2^ = 0.002), as revealed by the second one-way ANCOVA. The partial η^2^ indicated a very small effect size according to Cohen [61]. Bonferroni-corrected post-hoc analysis indicated that women with gPTSD rated all modes of service provision more favourably than women with CB-PTSD (*p* = 0.020, *M*_*Diff*_ = 1.073, 95% CI [0.126, 2.020]), as shown in Table 8. No significant effects were observed for the other groups.

**Table 8:** Group differences in the total sum score of preferences for mode of service provision

(I) Comparing symptom groups	(J) Comparing symptom groups	Mean difference (I-J)	*p*	95% CI	
				LL	UL
without^a^	CB-PTSD	.389	.371	-.216	.994
	gPTSD	-.684	.088	−1.435	.067
CB-PTSD	without^a^	-.389	.371	-.994	.216
	gPTSD	**−1.073**	.020*	−2.020	-.126
gPTSD	without^a^	.684	.088	-.067	1.435
	CB-PTSD	**1.073**	.020*	.126	2.020

*CI* Confidence interval, *LL* Lower limit, *UL* Upper limit

Significant group mean differences are printed in bold

^a^without clinically significant symptoms of postpartum PTSD

^*^p < .05

#### Subscales

A one-way MANCOVA revealed significant differences between groups in their preference ratings for the subcategories of service provision mode (F(4, 7550) = 4.082, *p* = 0.003, partial η^2^ = 0.002, Wilk's Λ = 0.996) with very small effect sizes according to Cohen [61]. Bonferroni-corrected post-hoc tests indicated that the gPTSD group preferred both direct and delayed modes of communication more than the CB-PTSD (*p* < 0.001, *M*_*Diff*_ = 0.654, 95% CI [0.234, 1.075]; *p* = 0.049, *M*_*Diff*_ = 0.696, 95% CI [0.002, 1.389]) and the group of women without clinically relevant postpartum PTSD symptoms, (*p* = 0.009, *M*_*Diff*_ = 0.441, 95% CI [0.080, 743]; *p* = 0.040, *M*_*Diff*_ = 0.565, 95% CI [0.018, 1.112]), see Table 9. At *p* = 0.049, the difference in preference for delayed communication modes between the gPTSD group and the CB-PTSD group should be considered borderline significant and requires careful interpretation [62].

**Table 9:** Group differences in the sub scores of preferences for direct and delayed modes of service provision

(I) Comparing symptom groups	(J) Comparing symptom groups	Mean difference (I-J)	*p*	95% CI	
				LL	UL
Direct communication					
without^a^	CB-PTSD	.243	.096	-.028	.514
	gPTSD	**-.411**	.009**	-.743	-.080
CB-PTSD	without^a^	-.243	.096	-.514	.028
	gPTSD	**-.654**	< .001***	−1.075	-.234
gPTSD	without^a^	**.411**	.009**	.080	.743
	CB-PTSD	**.654**	< .001***	.234	1.075
Delayed communication					
without^a^	CB-PTSD	.131	1,000	-.316	.578
	gPTSD	-.565^*^	.040*	−1.112	-.018
CB-PTSD	without^a^	-.131	1.000	-.578	.316
	gPTSD	**-.696**	.049*	−1.389	-.002
gPTSD	without^a^	**.565**	.040*	.018	1.112
	CB-PTSD	**.696**	.049*	.002	1.389

*CI* Confidence interval, *LL* Lower limit, *UL* upper limit

Significant group mean differences are printed in bold

^a^without clinically significant symptoms of postpartum PTSD

*p** < **.05. **p < .01 ***p < 0.001

## Discussion

The aim of this study was to explore the preferences for support of women with mental health problems in the postpartum period. The study specifically explored whether women's preferences for treatment and counselling services as well as the mode of service provision varied depending on whether they experienced symptoms of CB-PTSD, gPTSD, or were without clinically relevant symptoms of postpartum PTSD. Understanding these preferences is crucial given the increasing recognition of the need for personalized care in mental health support for women experiencing postpartum PTSD.

Most women preferred support from professional and personal confidants (e.g.(family-)midwives, family member, friend, colleague). Women with CB-PTSD showed less preference for all treatment and counselling services, and psychotherapeutic services in particular, compared to women without postpartum PTSD symptoms. All women preferred direct communication modes (e.g., in person, video conference) over delayed ones (e.g., chat, e-mail). Women with gPTSD had a stronger preference for both direct and delayed modes of service provision compared to women with CB-PTSD and women without postpartum PTSD symptoms.

### Preference for treatment and counselling services

Midwives and family midwives were the most preferred options for treatment and counselling for mental health issues for women in the postpartum period across all symptom groups. While previous qualitative studies have already identified midwives as a highly relevant professional group for women with postpartum PTSD [63, 64], our finding is essential as it provides the first conclusive quantitative data on this issue. In a previous study based on the INVITE study, which examined the treatment and counselling preferences of women with and without PPD and PAD within a smaller data set, it was also found that (family-)midwives were the most popular choice for support among women [37]. These findings indicate that (family-)midwives are a crucial resource for treating and counselling women with a range of postpartum mental health problems. Midwifery care is a legal right for women in Germany during pregnancy, during labour, and up to 12 weeks after birth [65]. Further and more comprehensive care can be provided by family midwives up to the child's first birthday [66]. This may include advising women and the family on child rearing, connecting families with other support services, and accompanying them to the authorities. The ongoing support that (family-)midwives can offer in this way, seems to make them trusted confidants that women prefer to approach when struggling with postpartum mental health problems.

Our study found that women preferred turning to confidants from a professional context (i.e. (family-)midwives) as a source of postpartum support even slightly more than those from their personal environment (family members, friends, and colleagues) and women in the same situation. Those personal sources of support ranked as the third and fourth most popular sources of support. Previous research already showed that women experiencing mental health problems in the postpartum period seek support from confidants within their personal environment and that peer support offers relief [31–33, 67], so the high preference found in our study is not surprising. What is notable is that no previous studies have determined whether women preferred the advice from these personal confidants over professional sources. We were able to show that the women in our study slightly preferred professional confidants over their personal confidants. Given that the highest rated barrier to help-seeking for women with and without postpartum PTSD in another study of our team was ‘I would rather talk to friends or family about my problems’ [68], it is noteworthy that we found a preference for professional help from (family-)midwives over family, friends, etc. This underlines midwives’ affiliation with what women perceive as their trusted confidants. It seems reasonable to suggest that one factor for the preference for (family-)midwives as professionals is that women may be concerned about being stigmatised by their private environment when disclosing their problems, given that birth is generally viewed by society as a joyous event.

Besides (family-)midwives as most favoured for treatment and counselling, gynaecologists, were ranked as the fifth most popular service option just after the personal confidents. Like (family-)midwives, gynaecologists tend to have a long, consistent relationship with their patients, following them through pregnancy and the postpartum process, therefore making them another trusted source of support. It should be noted that the long relationship between gynaecologists and patients is typical of the German healthcare system and may not be applicable to the healthcare context in other countries.

In terms of psychotherapeutic services, women had a clear preference for outpatient clinics (e.g. psychiatric, psychosomatic, or psychotherapeutic outpatient clinics and specialized trauma outpatient clinics) over day clinics or inpatient clinics. This may reflect women's concern about being separated from their children during hospitalisation, a situation mothers typically seek to avoid [69], probably further due to increased feelings of guilt, shame, fear of appearing as a bad mother, or concerns that their child might be taken away. In line with this, women with CB-PTSD, gPTSD, and women without clinically relevant postpartum PTSD symptoms also showed a preference for mother–child therapy over therapy for the mother alone for psychotherapeutic treatment options. However, this trend was less evident in the CB-PTSD group and the gPTSD group and even reversed in women comorbid with CB-PTSD and gPTSD. This may suggest that women who experience high distress due to postpartum PTSD symptoms may prefer to receive therapy for themselves without the child. This gives rise to the question of how to identify appropriate childcare for women who are affected. The integration of individual therapy with interventions targeting the mother–child bond in a day-clinic setting where childcare is secured has been demonstrated to yield substantial benefits for maternal mental health treatments [70].

#### Group differences in the preference for treatment and counselling services

Women with CB-PTSD had a significantly lower preference for all treatment and counselling services compared to women who did not have clinically relevant postpartum PTSD symptoms. This may stem from core PTSD symptoms such as the avoidance of trauma-related thoughts and feelings [8], as seeking treatment often involves confronting the traumatic event, which can trigger such thoughts and feelings [71, 72]. Since the group difference in lower preference for treatment and counselling services was only found for women with CB-PTSD compared to women without clinically relevant postpartum PTSD symptoms, but not for women with gPTSD, it is important to consider the unique characteristics of CB-PTSD compared to gPTSD: Avoidance of specific people, places, or situations that trigger trauma-related memories is another key symptom of PTSD [8], and may influence treatment preferences more in CB-PTSD than gPTSD. For women with CB-PTSD, the support system, especially services affiliated with the hospital where they gave birth, may be an (indirect) representation of the trauma itself. While support services are typically expected to provide help, these encounters may instead recall the traumatic birth, leading women with CB-PTSD to avoid such services, reducing their overall preference.

Moreover, women with CB-PTSD in our study were in the early stages of their disorder. The traumatic event of childbirth occurred between 6 and 26 weeks ago. It should be noted that the time since the traumatic event was not assessed for women with gPTSD. However, as these women can have experienced the traumatic event over the course of their entire lives, it is probable that it occurred longer ago, on average. It is therefore possible that the gPTSD group has already developed a greater readiness to disclose the trauma when turning to treatment or counselling services, which often takes years after the initial onset of the disorder [73]. In addition to the potential reluctance to engage in treatment and counselling, women with CB-PTSD may be particularly concerned about the possibility of being stigmatised in these settings as their trauma results from an event that society perceives as positive [68]. This may further explain why they may feel less comfortable disclosing such information, which is associated with less positive attitudes towards treatment and counselling services [68, 74].

Group comparisons of preferences for specific service categories revealed that women with CB-PTSD had a lower preference for psychotherapeutic services compared to women without clinically relevant postpartum PTSD symptoms. It is plausible that the aforementioned factors, which explain the lower overall preference of the CB-PTSD groups, may be most pronounced in the context of psychotherapeutic treatment. Firstly, the fear of re-experiencing traumatic content in psychotherapeutic treatment may be particularly pronounced in CB-PTSD women. Expert guidelines for psychotherapeutic treatment recommend trauma-focused approaches that directly address traumatic memories or related thoughts and feelings [75]. Furthermore, the utilisation of psychotherapeutic services necessitates the recognition of undergoing psychological distress. There is evidence supporting the hypothesis that the recognition of psychological distress influences attitudes towards help-seeking in women with postpartum PTSD. A study found that women with CB-PTSD who self-recognised their symptoms reported fewer barriers overall and fewer barriers from health beliefs. In gPTSD, recognition of PTSD was not a significant predictor of barriers [18]. The recognition and admission of mental health issues may prove even more challenging for women with CB-PTSD, given that these issues are associated with childbirth, which is traditionally viewed as a happy event. Consequently, women may feel reluctant to choose psychotherapeutic services due to concerns about being stigmatised [17].

### Preference for mode of service provision

In terms of preferences for mode of service provision, most women showed a clear preference for in person communication over all other options which is in line with previous research [46, 47, 76]. Direct modes of communication (i.e. video conference and telephone call) were preferred by all women over delayed communication modes (i.e. via chat, e-mail, chat, or app-based). Videoconferencing offers the most resembling setting to a face-to-face modality, as it also permits real-time interaction, including the use of visual cues and non-verbal communication. These elements help to build an intimate and personal connection which is important when discussing sensitive, even stigmatised issues such as postpartum PTSD. Although it may not achieve the same level of therapeutic alliance, patients appear to be generally satisfied with videoconferencing therapy [77]. Besides, it is possible that the increased use of videoconferencing in recent years has increased familiarity and confidence in it, which may be reflected in the results. In contrast, other web-based options appear to be less well known at present, which raises issues of confidentiality [78] and may explain why they were less popular in our study. In addition, safety concerns may be an important factor in preference for any telehealth service when discussing sensitive issues, for instance women who have reported IPV and wish to receive counselling from home via videoconferencing. Future research should look more closely at how safety concerns influence patient preferences.

#### Group differences in the preference for modes of service provision

Overall, women with gPTSD preferred all modes of service provision more than women with CB-PTSD or women without postpartum PTSD. Subsequent comparisons of preferences for the categories of modes of service provision revealed that women with gPTSD rated both direct and delayed communication more favourably than women with CB-PTSD and women without clinically relevant postpartum PTSD symptoms. It can be concluded that the preference for one mode of communication over another is not unique to a specific group, but rather, that the gPTSD group exhibits a greater preference for all available modes of service provision, both in general and across both categories. This suggests that group differences are not about individual characteristics of service provision modes, but rather represent a general openness to all modes of service provision. The openness to seek help in various modes of service provision can be seen a part of the willingness to seek help, which also depends on the extent to which those affected feel impaired by the symptoms in their daily lives [79]. If we assume that women with gPTSD have been experiencing symptoms for a longer period of time, in conjunction with a greater willingness to seek help, this may explain why they are more open to all modes of service provision. In contrast, women with CB-PTSD are usually in the early stages of the disorder and may not have reached the point where they recognise they need treatment or counselling, a process that can take years for mental health problems [80]. As a result, they may not have considered the different modes of service provision and may be more sceptical about them, as may be those who are without clinically relevant postpartum PTSD symptoms. This would imply that preferences for modes of service provision are less symptom-dependent and more dependent on one’s willingness and openness to treatment, which increases with the burden of disorder over longer duration of symptoms. This is consistent with the results of a study that analysed the preferences for mode of service provision of women with PPD, PAD, and women without clinically relevant postpartum PTSD symptoms, and found them to be independent from symptom groups [37]. Our study now shows that differences in the preferences of mode of service provision do exist between women with gPTSD and women with CB-PTSD as well as women without clinically relevant postpartum PTSD symptoms. Whether this is related to the presumed greater willingness to seek help due to a longer duration of symptoms needs further investigation.

### Strengths and limitations

Our study is the first to provide comprehensive, quantitative data on treatment and counselling preferences of women with postpartum PTSD and women without clinically relevant symptoms. The large sample size, achieved through a targeted recruitment strategy with a high response rate, provides valuable important insights that can inform the development of women-centred care for postpartum PTSD. Data were collected through telephone interviews, which are more personal than e.g. online surveys and present an appropriate method of data collection when dealing with sensitive issues [81]. A major strength is the differentiated consideration of the two subgroups of postpartum PTSD: CB-PTSD and gPTSD. This differentiation allows specific targets to be set for improvements in clinical practice. The decision to refer to a stressful experienced birth event for the CB-PTSD group allowed all women with clinical symptoms of CB-PTSD to be included. It should be noted that this resulted in a higher prevalence rate in our sample than in comparable samples in Germany [50]. At the same time, the prevalence in our sample is below the global prevalence of CB-PTSD and far below the prevalence of gPTSD in other samples [6, 15]. This could be related to the fact that our sample is considered a rather low-risk sample: Despite the broad recruitment strategy employed, the sample was characterised by above-average levels of education and income, with the majority of women having been born in Germany. The results should therefore not be generalised to women with a lower level of education, income, or a migration background. Another limiting factor of this study is that it was partially conducted during the COVID-19 pandemic. During the associated lockdowns, some treatment and counselling services were inaccessible, which may have influenced the preferences women reported in our study. Furthermore, the reliabilities of the self-constructed questionnaires for medical services (ω = 0.66) and professional and personal confidants (ω = 0.44) were questionable and unacceptable, respectively. The results should therefore be treated with caution. Finally, not all assumptions (i.e. normal distribution, linearity, homogeneity of regression slopes, and homogeneity of covariances) were fully met for each analysis. Despite the use of robust models and the incorporation of interaction terms to address violations of assumptions, it is possible that the results were still affected.

### Research implications

Future studies should endeavour to include more diverse samples, encompassing individuals from varying income and educational levels, migration backgrounds, sexual orientations, and gender identities. This is because individuals with lower income, lower education, a background of migration, non-heterosexual sexual orientation, and non-cisgender identities have the least access to care in the postpartum period [82–86]. Consequently, their preferences should be given greater consideration.

Furthermore, it is recommended that the study results be replicated, given that the findings of our study had very small effect sizes. In addition, it would be interesting to conduct the study with high-risk or inpatient samples to determine whether the observed effects are more pronounced in severe cases of postpartum PTSD and severe impairment. This should also include women with comorbid CB-PTSD and gPTSD, which was not possible in our study due to the small sample size of the comorbid group.

Moreover, the hypothesis that the higher preference for modes of service provision in the gPTSD group is due to a greater openness to treatment associated with a longer duration of symptoms requires empirical investigation. In this context, it would be worth to analyse previous experiences with treatment and counselling services and modes of service provision and their influence on preferences, since associations have been found for women with postpartum PTSD [87]. In the case of CB-PTSD, greater knowledge of healthcare services was associated with increased barriers among women with CB-PTSD, while greater knowledge of healthcare services among women without clinically relevant postpartum PTSD symptoms was found to be associated with decreased barriers [18]. The implications of these findings for treatment and counselling preferences remain to be elucidated.

### Practice implications

Based on the results of our study, we recommend to expand the care provided by (family-)midwives in Germany, as women with postpartum PTSD but also women without clinically relevant postpartum PTSD symptoms as well as women with PAD and PDD prefer receiving support from them the most [37, 88]. Midwives themselves recognise the need to expand their services to meet women's needs [89]. Expanding their services should therefore be supported by policy makers through increased efforts and funding. Furthermore, it is crucial to enhance the awareness of postpartum PTSD among (family-)midwives, ideally as part of the ongoing academisation of the midwifery profession in Germany [90]. The second highest preference for receiving support was from family members, friends, colleagues, and women in the same situation, which suggests that it would also be useful to raise the awareness of postpartum PTSD in the general population [91], thereby reducing the stigma that prevents especially women with CB-PTSD from seeking help [68]. Given that women with CB-PTSD showed a lower preference for all services, it is necessary to target this group in particular to facilitate their access to appropriate support services. Knowing the high prevalence of traumatic birth experiences and the fact that postpartum women usually have frequent contact with health professionals (midwives, gynaecologists, paediatricians) [25], these professionals should implement more standardised screenings for CB-PTSD and refer women at risk to appropriate support services. Referrals to appropriate services ought to consider the lower preference for psychotherapeutic services among women with CB-PTSD found in our study. For instance, it might be beneficial to provide more detailed information on the effectiveness of psychotherapeutic interventions [21], while taking possible concerns about disclosing their traumatic experience and the fear of stigma seriously. Another important finding of our study is the consistent preference for mother-baby interventions across groups. This underscores the need for a relational, systematic approach in perinatal healthcare.

In terms of modes of service provision, it is important to recognise the strong popularity of direct communication in an in person setting. However, direct communication via video conference appears to be a popular alternative for women. The fact that many barriers could be reduced by implementing telehealth features makes it worthwhile to expand their use in treatment and counselling contexts. Digital midwifery care, for instance, has already been demonstrated to be well received by postpartum women [92]. Other web-based modes of service provision (i.e. via e-mail, chat, or app-based) appear to be less popular with women. The objective here is to work with practitioners to identify potential applications of these web-based modalities and to foster familiarity among women.

## Conclusions

This study is the first to examine preferences for treatment and counselling services as well as modes of service provision among women experiencing postpartum PTSD symptoms. (Family-)midwives emerged as the most preferred treatment and counselling option across all groups. However, notable differences were observed between women with CB-PTSD and gPTSD. Women with CB-PTSD exhibited a significantly lower preference for all service options, particularly psychotherapy, compared to women without clinically relevant postpartum PTSD symptoms. This suggests potential barriers to treatment engagement for this group. In contrast, women with gPTSD demonstrated a broader acceptance of service provision modes compared to the other groups, suggesting more flexibility in how they access care. These differences underscore the need for tailored approaches in recognizing, screening, and treating postpartum PTSD. Specifically, women with CB-PTSD may require additional support to overcome treatment hesitancy, which could stem from factors such as fear or stigma. Further research is necessary to clarify how variables like duration of symptoms and prior treatment experiences impact these preferences. To bridge the treatment gap, it is crucial that future service designs consider these group differences, ensuring accessible and effective care for all women affected by postpartum PTSD.

## Data Availability

The dataset presented in this article is not publicly available because of legal and ethical constraints. Public sharing of participant data was not included in the informed consent of the study. Requests to access the datasets should be directed to the project managers and principal investigators Susan Garthus-Niegel or Julia Schellong.

## Footnotes

1 Family midwives are part of the German support system to improve care for vulnerable families, such as single mothers. Their work, primarily through home visits, extends from pregnancy to the child's first year and involves close collaboration with other health and social services. Unlike community midwives, who provide standard maternity care to a wider population, family midwives focus specifically on families who need additional support.
